# Applications of Federated Learning in Mobile Health: Scoping Review

**DOI:** 10.2196/43006

**Published:** 2023-05-01

**Authors:** Tongnian Wang, Yan Du, Yanmin Gong, Kim-Kwang Raymond Choo, Yuanxiong Guo

**Affiliations:** 1 Department of Information Systems and Cyber Security The University of Texas at San Antonio San Antonio, TX United States; 2 School of Nursing The University of Texas Health Science Center at San Antonio San Antonio, TX United States; 3 Department of Electrical and Computer Engineering The University of Texas at San Antonio San Antonio, TX United States

**Keywords:** decision support, distributed systems, federated learning, health monitoring, mHealth, privacy

## Abstract

**Background:**

The proliferation of mobile health (mHealth) applications is partly driven by the advancements in sensing and communication technologies, as well as the integration of artificial intelligence techniques. Data collected from mHealth applications, for example, on sensor devices carried by patients, can be mined and analyzed using artificial intelligence–based solutions to facilitate remote and (near) real-time decision-making in health care settings. However, such data often sit in data silos, and patients are often concerned about the privacy implications of sharing their raw data. Federated learning (FL) is a potential solution, as it allows multiple data owners to collaboratively train a machine learning model without requiring access to each other’s raw data.

**Objective:**

The goal of this scoping review is to gain an understanding of FL and its potential in dealing with sensitive and heterogeneous data in mHealth applications. Through this review, various stakeholders, such as health care providers, practitioners, and policy makers, can gain insight into the limitations and challenges associated with using FL in mHealth and make informed decisions when considering implementing FL-based solutions.

**Methods:**

We conducted a scoping review following the guidelines of PRISMA-ScR (Preferred Reporting Items for Systematic Reviews and Meta-Analyses Extension for Scoping Reviews). We searched 7 commonly used databases. The included studies were analyzed and summarized to identify the possible real-world applications and associated challenges of using FL in mHealth settings.

**Results:**

A total of 1095 articles were retrieved during the database search, and 26 articles that met the inclusion criteria were included in the review. The analysis of these articles revealed 2 main application areas for FL in mHealth, that is, remote monitoring and diagnostic and treatment support. More specifically, FL was found to be commonly used for monitoring self-care ability, health status, and disease progression, as well as in diagnosis and treatment support of diseases. The review also identified several challenges (eg, expensive communication, statistical heterogeneity, and system heterogeneity) and potential solutions (eg, compression schemes, model personalization, and active sampling).

**Conclusions:**

This scoping review has highlighted the potential of FL as a privacy-preserving approach in mHealth applications and identified the technical limitations associated with its use. The challenges and opportunities outlined in this review can inform the research agenda for future studies in this field, to overcome these limitations and further advance the use of FL in mHealth.

## Introduction

### Background

Mobile health (mHealth) generally refers to the use of mobile and wearable devices (eg, smartphones and smartwatches) in a health care setting [1]. In mHealth, health care–related data are typically collected from digital devices, such as biomedical sensors attached to the user’s body or portable devices with relevant applications installed [1]. More recently, there has been significant interest in using mHealth technologies for the collection of patient-reported outcome measures as such measures can enhance communication between patients and health care providers [2]. Additionally, machine learning (ML) techniques have been used to enhance diagnostic precision and facilitate remote, fine-grained, and high-quality health care in the mHealth field [3-5]. However, the traditional approach of training ML models requires centralized datasets, where a central server has access to the data of all patients. This can create privacy concerns for patients who may not want to share their personal information, and the regulatory requirements within the health care industry may also limit the sharing of sensitive information [6]. As a result, this leads to the existence of data silos, which will limit the use of traditional ML-based solutions. Furthermore, the limitations on data availability caused by privacy concerns and data silos can impede the implementation of large-scale health care systems, introducing biases in ML-based approaches. Consequently, these challenges can exacerbate the existing health disparities [7].

Federated learning (FL) is a potential solution for mitigating the above-discussed challenges associated with sharing of raw patient data in mHealth applications. FL is a ML approach where the trained model parameters, rather than the raw data, are shared during the learning process [8]. FL-based methods typically involve a central server that holds a global model, and during each round of FL, a random subset of users is chosen to participate in the training of the global model. The selected users train the models locally using their data and then send their trained models back to the server. The server then aggregates the models and updates the global model, and the process repeats for subsequent FL rounds. However, it is worth noting that most existing FL frameworks in the health care domain are not designed to support data from mobile and wearable devices, which are increasingly important sources of data, given their widespread usage [9].

### Objective

Although FL is a viable tool to support privacy-preserving health care decision-making, its implementation in clinical practice is not straightforward and has several limitations. Therefore, this scoping review aims to explore the current progress of FL-based mHealth applications (eg, how can FL be used to deal with sensitive, unbalanced, and heterogeneous data across users’ mobile devices?). Furthermore, this review discusses the associated limitations and challenges, providing health care professionals and policy makers with essential information for informed decision-making regarding the adoption of FL-based solutions in healthcare.

In summary, this review seeks to achieve the following objectives: (1) present an overview of FL applications in mHealth settings, (2) assist practitioners and policy makers in understanding the challenges of implementing FL in mHealth settings, and (3) explore and identify potential approaches for developing efficient, effective, and robust FL systems for mHealth applications.

## Methods

### Overview

This scoping review was prepared and reported according to the guidelines of the PRISMA-ScR (Preferred Reporting Items for Systematic Reviews and Meta-Analyses Extension for Scoping Reviews) framework [10].

### Data Sources and Search Strategy

Our literature search was conducted using several databases, such as PubMed, JMIR, Web of Science, IEEE Xplore, ACM Digital Library, ScienceDirect, and Springer, for articles published between January 2015 and January 2022. The initial search was carried out using keyword combinations such as “federated learning” AND “mobile” AND “health*” to identify papers published between January 2016 and January 2022 in all 7 databases. The abstract was used to extract the necessary information and avoid selection bias. A second search was also carried out using keyword combinations, such as “collaborative learning” AND mobile AND health* to identify articles published between January 2015 and January 2018 in PubMed, JMIR, Web of Science, IEEE Xplore, and ACM Digital Library. This second search was conducted because FL was first coined by Google in 2016, and collaborative learning was the predecessor and used interchangeably with FL. The details of the search results for each search engine are presented in Multimedia Appendix 1.

### Inclusion and Exclusion Criteria

This study focuses on the use of FL in mHealth rather than the broader eHealth settings. Specifically, it examines how FL can be used with data captured by mobile devices to solve real-world health care problems. As such, articles that developed new FL techniques but were not specifically designed for mHealth applications were excluded. Additionally, only articles published in refereed journals and conference proceedings were considered, whereas other types of publications, such as conference abstracts only, books, editorials, and commentaries were excluded.

### Study Selection

**Figure 1:**
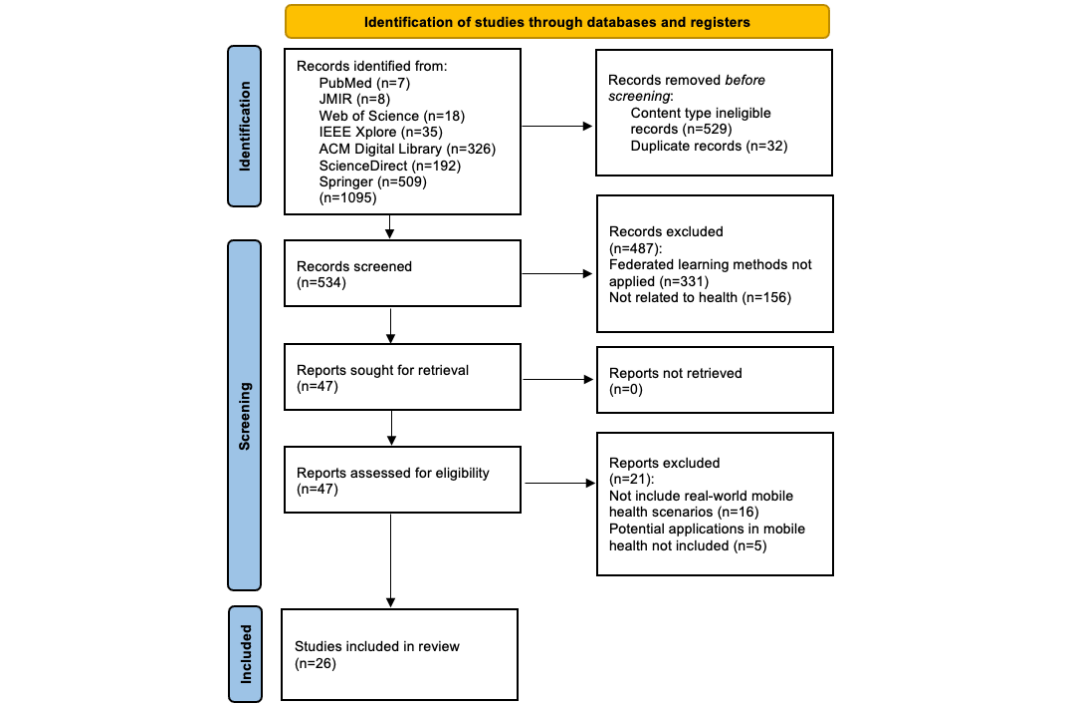
PRISMA (Preferred Reporting Items for Systematic Reviews and Meta-Analyses) 2020 flow diagram of the study selection process.

The study selection process involved the retrieval of 1095 publications through a database search, followed by the removal of 529 studies using the content-type filter in each database and 32 duplicate studies. Out of the remaining 534 studies, 487 were excluded after title and abstract screening, resulting in only 47 relevant articles. A final screening process was conducted, and 26 of the 47 articles were found to meet the inclusion criteria and included in the review. Two authors independently screened the titles and abstracts of the identified studies to determine their eligibility for this scoping review based on the above inclusion and exclusion criteria. Minor disagreements between the reviewers were resolved, and the reviewers reached an agreement. The process of study selection is illustrated in Figure 1.

## Results

### Search Results

Of the 1095 articles located, only 26 were chosen for further analysis. These 26 publications were categorized into the following 2 themes: remote monitoring and diagnostic and treatment support based on their potential applications in mHealth. The 2009 United Nations Foundation and Vodafone Foundation report [11] presented 7 application categories within the mHealth field, and our included studies are related to 2 of these areas that are remote monitoring and diagnostic and treatment support. Hence, the included papers were separated into these 2 categories.

### Applications and Type of Data Used

In mHealth settings, 1 application area of FL is remote monitoring, which includes monitoring self-care abilities, health status, and progression of chronic diseases. Twelve of 26 studies specifically focus on recognizing human activities using mobile sensors with FL, with a focus on distinguishing different activities of daily living (ADLs), such as walking, sitting, and standing. Recognizing ADLs is particularly useful for monitoring senior citizens’ self-care abilities and health status [5]. Two studies investigated the potential of using mobile sensor signals to monitor Parkinson disease by analyzing specific activities, such as arm droop, balance, and gait [12,13]. It was also noted that patients with Parkinson disease frequently experience freezing of gait, which can be easily detected in their daily activities [14]. Another disease that can be monitored in daily life is epilepsy. For example, Gong et al [15] used a data set comprising electrocorticography recordings of patients with epilepsy to facilitate the monitoring of such patients in their daily activities. Three studies [16-18] demonstrated the use of FL in monitoring stress using data collected from mobile devices during certain activities. Two types of data commonly used in these studies are electrocardiograms (ECGs) and electrodermal activity. Additionally, 3 other studies [19-21] proposed FL-based systems for cardiac health monitoring. For instance, Raza et al [21] designed an FL framework for ECG monitoring, which has the ability to effectively classify various arrhythmias. Furthermore, the authors incorporated an explainable artificial intelligence–based module on top of the classifier to ensure the interpretability of the classification results, thereby enabling clinical practitioners to better understand the prediction results. There are various other applications as well. Gong et al [22], experimentally evaluated their proposed collaborative learning scheme on diabetes data set and demonstrated its practicality for mHealth monitoring scenarios. Siddiqui et al [23] integrated FL with the Internet of Medical Things architecture to detect the risk of obesity in individuals. BMI data were analyzed to assess the obesity risk, and expert recommendations were generated based on the results.

Another common application area is diagnostic and treatment support. For example, the diagnosis of mental health disorders currently relies heavily on the subjective judgment of the doctor through communication with patients and the responses from the patient health questionnaires [9]. In this regard, Xu et al [9] proposed a federated depression detection method. The evaluations were conducted in a hospital setting, with participants each receiving a smartphone to collect data on keyboard usage during specific sessions. Additionally, data from participants’ weekly tests, such as the Hamilton Depression Rating Scale [24] and the Young Man Mania Scale [25], were also collected. There have also been efforts to use FL for detecting skin diseases [26,27], and other medical conditions, such as cancer [28]. For example, Wu et al [27] developed an on-device federated contrastive learning framework for dermatological disease diagnosis with limited labels. Guo et al [28] proposed an FL-based system and experimented on a breast cancer data set by simulating mobile scenarios and demonstrating the potential of future mHealth applications for cancer detection. Moreover, Sun et al [29] proposed an FL framework for perioperative complications prognostic prediction, which demonstrates the potential of FL in providing treatment support. The proposed framework was tested using experiments on a real-world mHealth data set and the results suggested the utility of the proposed framework. A summary of the applications can be found in Table 1.

Most of the studies (14 of 26) used human activity recognition data sets, often collected using smartphones and smartwatches. The Inertial Measurement Unit, which comprises accelerometers, gyroscopes, and magnetometers, is another commonly used sensor type in activity recognition. The most common data types for ADLs recognition are acceleration, angular velocity, and magnetometer. In studies on Parkinson disease, acceleration data is used to detect freezing of gait as it is a symptom unique to individuals with the condition [13]. Moreover, a data set is used by Chen et al [12] to record acceleration and angular velocity during various activities, such as arm droop, balance, gait, postural tremor, and resting tremor. Other medical conditions, such as epilepsy, can also be detected through activity monitoring, with electrocorticography being used to classify patients with epilepsy [15]. Other data types, such as ECG, electrodermal activity, blood volume pulse, and body temperature, can be used to monitor stress [16-18], cardiac health [19-21], and related conditions. Skin disease or breast cancer detection often relies on the analysis of disease-related images [26-28]. Detailed information about the data sets and data types used in the included studies can be found in Multimedia Appendix 2.

**Table 1:** Applications of federated learning in mobile health.

Areas and applications			Studies
**Remote monitoring^a^**			
	Activities of daily living	[4,12,13,30-38]	
	Parkinson disease	[12,13]	
	Epilepsy	[15]	
	Diabetes	[22]	
	Cardiac health	[19-21]	
	Obesity	[23]	
	Stress	[16-18]	
**Diagnostic and treatment support^b^**			
	Depression	[9]	
	Dermatological disease	[26,27]	
	Cancer	[28]	
	Perioperative complications	[29]	

^a^Remote monitoring allows individuals to self-monitor their health status and manage the progression of chronic diseases from a remote location, such as their own home.

^b^Diagnostic and treatment support refers to the provision of assistance for the diagnosis and treatment of patients from a remote location.

### Challenges for FL-Based Applications in mHealth Settings

#### Overview

The use of FL in mHealth settings is not without challenges, and examples include statistical heterogeneity, system heterogeneity, expensive communication, privacy leakage, and real-time data stream. These challenges were chosen for analysis because statistical heterogeneity, system heterogeneity, and expensive communication are FL-specific challenges, whereas real-time data stream is a crucial aspect of real-world mHealth applications. Table 2 provides a summary of the challenges identified in the selected studies. It should be noted that data scarcity and scalability are also challenges identified in the selected papers, but they are not specific to FL or mHealth, and therefore, will not be discussed further in this paper.

**Table 2:** Challenges for federated learning (FL)–based applications in mobile health.

Challenges	Description	References
Statistical heterogeneity	Users’ data can be highly nonidentically distributed, characterized by diverse, imbalanced, and heterogeneous patterns, which can pose challenges for training models.	[4,12,16,18,21,30,31,34-38]
Expensive communication	An increase in the number of devices participating in an FL system would result in an increase in the number of global communication rounds, as well as the size of the transmitted messages in each round (ie, communication is typically slower than local computation in FL).	[4,9,12,13,15,20,21,26-28,30,32,34-38]
System heterogeneity	The storage, computational, and communication capabilities of each device may differ due to variability in hardware and network connectivity, leading to system incompatibility.	[16,34,35]
Privacy leakage	Communicating model updates throughout the training process can nonetheless reveal sensitive information, so the privacy of FL needs to be enhanced.	[12,15,16,21,22,28,29,33,37,38]
Real-time data stream	Sensing measurements are generated continuously, and the FL process should be working continuously with new emerging data.	[9,21,23,35,36,38]
Data scarcity	Labeled data are usually limited because it is costly and time-consuming to obtain labeling feedback regarding sensing data.	[21,22,33,34]
Scalability	As the amount of data available for training and the complexity of models increase, the resources required to train and run these models also increase.	[17,20,33,38]

#### Statistical Heterogeneity

In typical FL systems, a significant number of user devices, such as smartphones, tablets, and smartwatches are used. These devices frequently generate and collect data in a nonidentically distributed (non-IID) manner, which can negatively impact the accuracy of the FL-based approach. Moreover, users’ data patterns are diverse, imbalanced, and heterogeneous, particularly when data recorded during user activities are considered.

#### Expensive Communication

In large-scale FL systems, the use of numerous user devices necessitates multiple rounds of global communication between users and the server. This can significantly impact the performance of the FL system, especially in resource-constrained environments such as mobile networks or Internet of Things devices [39]. This reinforces the importance of developing communication-efficient methods within FL systems, which aim to exchange minimal information as part of the training process, in order to reduce the communication cost of the system.

#### System Heterogeneity

In FL networks, the storage, computational, and communication capabilities of each device can vary widely due to diversity in hardware and network connectivity (eg, device types, operating systems, and storage capacity) [39]. These system-related constraints can result in system incompatibility due to data formats and there is a risk that only a small fraction of devices will be active in a federated network at any one time, or active devices may drop out at a given iteration [40]. As a result, system heterogeneity can have a considerable impact on the performance of FL-based approaches in practice.

#### Privacy Leakage

Although FL is intended to mitigate privacy concerns associated with conventional ML-based applications by only sharing model updates, such as gradient information instead of raw data, the communication of model updates throughout the training process may still reveal sensitive information, either to a third party or the central server [41]. Attempts have been made to design tools to enhance the privacy of FL using secure multiparty computation (SMC) [42] and differential privacy (DP) [41,43]. However, these methods can be costly and may negatively impact model performance or system efficiency [40]. Therefore, balancing the tradeoffs between privacy and performance is a significant challenge in the practical implementation of FL-based systems.

#### Real-Time Data Stream

Real-time data, such as sensing measurements, commonly occur in mHealth settings. Consequently, when using FL in mHealth settings, the FL model must be able to respond instantaneously to adjustments and updates in sensor data. In other words, the FL process must be able to operate continuously with incoming data streams in real-time to ensure that the model can adapt to and make predictions based on the most recent data.

### Solutions to Address Challenges

#### Overview

In the following paragraphs, we will provide an in-depth analysis of how the identified challenges can be overcome based on the findings from the articles included in our review (see Table 3). A summary of the included studies can be found in Multimedia Appendix 3.

**Table 3:** Solutions for addressing the challenges of federated learning–based applications in mHealth.

Challenges and solutions			References
**Statistical heterogeneity**			
	Meta-learning	[4]	
	Model personalization	[12,18,21,30,36]	
	User clustering	[34,35,37]	
	Adaptive update scheme	[16,31,38]	
**Expensive communication**			
	FedAvg	[4,9,12,13,20,26,27,30,36]	
	Flexible local updates	[15,37,38]	
	Compression schemes	[21,28,32,34,35]	
**System heterogeneity**			
	Active sampling	[16]	
	Fault tolerance	[34,35]	
**Privacy leakage**			
	Differential privacy	[28,38]	
	Homomorphic encryption	[12,15,22,37]	
	Strict information sharing scheme	[16,21,29]	
	2-stage privacy-preserving scheme	[33]	
**Real-time data stream**			
	Incremental learning	[36]	
	Web-based learning	[38]	
	Repeat working with continuously emerging data	[9,21,23]	
	Periodical update	[35]	

#### Statistical Heterogeneity

Twelve of the included studies addressed the challenge of statistical heterogeneity. For example, Ek et al [30] empirically evaluated 3 FL algorithms (ie, FedAvg [8], FedPer [44], and FedMA [45]) to determine their effectiveness. Findings from their evaluations suggested that the averaging of more personalized models leads to performance degradation when the learned global model is used for evaluation on the test set. Several studies have attempted to address this challenge by designing personalized FL methods for adapting global models for individual clients.

One possible approach is meta-learning, specifically the federated representation learning framework proposed by Li et al [4]. This framework used a signal embedding network, that is meta-trained in an FL manner and the learned signal representations were further fed into a personalized classification network for better activity prediction for each user. One of the most common approaches is model personalization, in which base layers and personalization layers are trained separately. For instance, in Chen et al’s [12] and Raza et al’s [21] studies, transfer learning was applied to learn personalized models for each user, as the higher layers aim at learning more user-specific features whereas the lower layers focus on learning common and transferable features. Furthermore, Liu et al [18] added a simple user embedding to the neural network, which was kept only on that user’s device. Wu et al [36] proposed a generative convolutional autoencoder network and fine-tuned the model parameters of higher layers of generative convolutional autoencoder to obtain more accurate personalized models. Another way to address statistical heterogeneity is user clustering, which enables the FL system to capture the underlying relationships between users as studied by Ouyang et al [34] and Tu et al [35]. By clustering users, those in the same group can collaboratively learn personalized models. Similarly, Xiao et al [37] designed a feature extractor capable of identifying and extracting the local features and global relationships from heterogeneous data to address statistical heterogeneity.

In addition to the abovementioned methods, several studies attempted to address statistical heterogeneity by designing update schemes to guarantee convergence for non-IID data. Traditional FL methods may diverge when the data are not identically distributed across devices, particularly when the chosen devices perform too many local updates [40]. Accordingly, Yu et al [38] derived a personalized strategy for semisupervised learning users who require personalized service. Specifically, the personalized models will be initialized using the general model downloaded from the server and users will additionally convey gradients to the server once during each training iteration to obtain personalized models. Gudur and Perepu [31] introduced a 2-version FL framework for addressing heterogeneity issues in non-IID scenarios by leveraging overlapping information gained across activities—one using model distillation update and the other using weighted update. Additionally, Zhang et al [16] developed a new local update scheme and an adaptive global update scheme, which jointly enable each device to decide the optimized local and global update strategies to deal with the non-IID problem.

#### Communication Efficiency

Of the 26 studies, 17 studies addressed the challenge of expensive communication. Among them, 9 studies [4,9,12,13,20,26,27,30,36] used the FedAvg algorithm [8] to reduce communication overhead, as FedAvg [8] is frequently adopted as a method to reduce the number of required communication rounds. In addition, Xiao et al [37] proposed a system that only calculates and circulates the average weights after receiving the model weights from a certain number of connected users in order to improve communication efficiency. Yu et al [38] developed an unsupervised gradients aggregation strategy together with FedAvg to decrease the communication overhead. Moreover, Gong et al [15] proposed a scheme based on the alternating direction method of multipliers [46] to decompose the logistic regression model into smaller subproblems that can be locally computed and reduce communication cost.

The previously mentioned studies focus on developing optimization methods that enable flexible local updates, thereby reducing the overall number of communication rounds. However, the model compression scheme can also ensure communication efficiency by significantly decreasing the amount of information communicated in each round. Notably, there are various methods for achieving this. For instance, Liu et al [32] developed a collaborative privacy-preserving learning system that mainly considered 2 different parameter exchange protocols—round-robin and asynchronous—both of which aim to ensure low communication cost. Ouyang et al [34] designed a learned cluster structure for their system, which allows the use of clusterwise straggler dropout and correlation-based node selection to reduce communication overhead. Moreover, Tu et al [35] proposed FedDL, which can reduce the number of parameters communicated between users and the server through a dynamic layerwise sharing scheme. This is because only the lower layers of local models need to be uploaded to the server for global training. In separate work, Raza et al [21] designed a framework for ECG monitoring and proposed a new method called layer selection, which can significantly reduce the overall communication cost. In addition to the aforementioned methods, Guo et al [28] offloaded health monitoring and model training tasks to private servers of hospitals with strong computing resources to reduce communication cost and protect the privacy of users.

#### System Heterogeneity

Three of the studies focused on system heterogeneity. For instance, Ouyang et al [34] leveraged the inherent relationships between users to dynamically drop nodes during the FL process, where the server will drop users who converge slower than others within the same cluster or are less related to others in the same cluster. Tu et al [35] used the similarity among users’ model weights to learn the layerwise sharing structure, which can be regarded as an asynchronous scheme that mitigates the impact of straggling users in heterogeneous environments. Furthermore, as proposed by Zhang et al [16], the bottom-up design of the new local update scheme and the adaptive global update scheme allows the FL system to meet device-specific optimization goals (eg, energy savings) while strictly protecting user privacy.

#### Privacy Leakage

In the studies analyzed, 10 of them tackled the issue of privacy leakage that may arise when implementing FL in real-world scenarios. There are 3 main ways to protect data privacy in the FL framework [40], which are as follows: SMC [42], DP [41,43], and homomorphic encryption [47]. Four of these studies [12,15,22,37] used homomorphic encryption to avoid information leakage. Two studies adopted DP. Specifically, Yu et al [38] leveraged DP in combination with the Byzantine-robust aggregation rule [48] to defend against malicious clients and prevent data recovery attacks, whereas Guo et al [28] proposed 2-stage strong privacy protection based on DP to resist both external and internal security risks. Other novel methods were also proposed. A 2-stage privacy-preserving scheme was developed [33] to deliver great recovery resistance to maximum a priori estimation attacks. Zhang et al [16] designed an abnormal health detection system that strictly prohibited any violations to meet the privacy requirements and the only information allowed to be sent to the server is the weight updates generated by locally trained models on each device. Raza et al [21] proposed a solution to enhance privacy by only sharing weights that contain more common and low-level (ie, less private) features. Sun et al [29] proposed a solution where the training process will not reveal any information due to the masked weighted sum, which was uploaded by users and would not cause information leakage.

#### Real-time Data Stream

Six of the studies considered the use of real-time data in mHealth. For example, 3 of these studies [9,21,23] developed methods that can continuously adapt to new emerging data by repeating the entire process with the accumulation of new data. Several studies developed specific methods or used unique data sources to address this issue. More specifically, the system proposed by Tu et al [35] can periodically update the layerwise sharing structure and models to deal with users’ dynamic data distribution. Yu et al [38] let unlabeled users be trained with the online learning method so that the proposed system can use only a small number of labeled users with limited samples to train a model with competitive performance along with the massive real-time stream sensing data produced by unlabeled users. The framework proposed in reference [36] was able to perform incremental learning [49], where both the cloud model and user models can be continuously updated when new user data is encountered. Furthermore, the learned cloud model that captures the generic information from users can be easily deployed as a previous model for new users.

## Discussion

### Principal Findings

To advance the understanding of the use of FL in mHealth, a scoping review of 26 published studies was conducted. The results of this review indicated that FL, a relatively new privacy-preserving method shows great promise for use in mHealth. The reason for this is that medical data often resides in isolated data silos, and privacy concerns can restrict access to this data. Without access to sufficient data, conventional ML is limited in its utility. FL offers a solution by supporting collaborative model training on data from different sources while preserving privacy. Additionally, the results of this scoping review identify the potential application domains for FL in mHealth and the challenges that must be addressed to fully realize its potential. Furthermore, the review highlights potential approaches to addressing these challenges and improving the effectiveness of FL in mHealth applications.

The first aim of this review is to summarize the applications of FL systems in mHealth. The findings indicate that FL can be used for remote monitoring and diagnostic and treatment support within the mHealth field. More specifically, it can be applied to address a wide range of real-world problems, such as Parkinson disease monitoring, cardiac health monitoring, and mental health disorders diagnosis. When using FL in mHealth, various types of sensor data can be collected, including acceleration, angular velocity, magnetometer, ECG, and temperature data, to address different problems.

In other words, FL is a paradigm shift from centralized data lakes, and the use of FL in mHealth has a significant impact on various stakeholders, including patients, clinicians, and health care providers. By implementing large-scale FL-based applications in mHealth, patients can benefit from improved clinical decision-making without having to compromise their privacy. With such systems, patients can receive better care even from their own homes, particularly for those with rare or geographically uncommon diseases. More importantly, the adoption of FL in mHealth can improve patient privacy and lower the hurdle to becoming a data donor. For clinicians and practitioners, using FL-based systems in mHealth can augment their expertise with knowledge from larger populations [50]. This can help to mitigate the issue of bias in clinical practice that occurs when clinicians are exposed to a subgroup of the population based on their location and demographic environment, which can lead to inaccurate assumptions about the probability of certain diseases or their interconnection [50]. By using FL-based systems, clinicians can ensure a consistency of diagnosis that is not currently attainable, improving the quality of care for patients. For hospitals, policy makers and health care providers, the stabilization of federated-based systems for mHealth can offer better services for diverse population groups without worrying about patient privacy concerns. In FL-based systems, patients can maintain full control and possession of their data with limited risk of misuse by third parties, which makes it a more secure and trustworthy option for patients.

Additionally, this scoping review aims to provide practitioners, policy makers, and health care providers with a comprehensive understanding of the challenges associated with using FL in mHealth. The review specifically focuses on issues, such as expensive communication overhead, statistical heterogeneity, system heterogeneity, privacy leakage, and real-time data stream. This review provides an in-depth examination of these challenges and highlights the potential effects they could have on FL systems implemented in real-world settings. Through the review, practitioners and policy makers can gain insight into the unique characteristics of implementing FL in mHealth and use those insights to make informed decisions when designing, implementing, and evaluating FL systems in mHealth.

This review’s final goal is to identify potential solutions that can be applied to address various challenges associated with the use of FL in mHealth applications. One of the main challenges identified in the literature is high communication cost, which can be addressed through methods, such as FedAvg and model compression schemes. Another major challenge is statistical heterogeneity, which can lead to significant performance degradation in FL systems that use traditional aggregation methods, such as FedAvg as this approach is sensitive to heterogeneous and imbalanced data distributions among users, resulting in decreased model performance. This highlights the need for more robust and personalized FL approaches that can adapt to the unique characteristics of each user’s data distribution. Data security is also a significant concern in FL for mHealth, and many studies have proposed the use of additional privacy protection mechanisms, such as DP and SMC. However, these mechanisms often result in reduced model performance or system efficiency [51], highlighting the need for further research to balance the trade-offs between privacy and system performance. Additionally, this review identifies system heterogeneity as another challenge in FL for mHealth, but to date, only a limited number of studies addressed this issue. In summary, further research is necessary to tackle these prevalent challenges and improve the performance of FL systems.

### Limitations

This review has several limitations. First, this review focused on 5 key challenges related to applying FL, whereas other challenges like data scarcity and scalability were not discussed due to page limit. Second, while the search period covered several years, the majority of the included studies were from 2020 to 2021. This could be due to the fact that FL was first introduced by Google in 2016, which has led to an increase in research on this topic in recent years. Fortunately, this allows for a more current understanding of FL’s development and impact on mHealth, and it is expected that more studies will be published in the coming years. Furthermore, for the paper collection process, the search terms should have certain limitations and were not able to include all relevant studies. However, the studies that were gathered through our data sources and search strategy should provide a comprehensive overview of the field.

### Conclusions

This scoping review provides valuable insights for different stakeholders on the potential benefits and challenges of using FL in mHealth. FL is a novel and efficient privacy-preserving learning method that can be applied in a wide range of fields, particularly in mHealth, where it has been proposed for various applications such as Parkinson disease monitoring, cardiac health monitoring, and mental health disorder diagnosis. However, many barriers prevent practitioners from effectively implementing FL in mHealth. The most common obstacles include communication costs, statistical and system heterogeneity, and privacy leakage. Policy makers and health care providers need to consider these challenges when designing and implementing FL systems. This will help them to provide better services and support for health professionals, patients, patients’ families, the public, and other relevant parties. Further research is needed to address these common challenges and improve the performance of FL systems.

## Appendix

Multimedia Appendix 1 Search strategy and search results for different databases.

Multimedia Appendix 2 Summary of datasets used in the selected studies.

Multimedia Appendix 3 Summary of the selected studies.

## Abbreviations

ADL: activity of daily living
DP: differential privacy
ECG: electrocardiogram
FL: federated learning
mHealth: mobile health
ML: machine learning
non-IID: nonidentically distributed
PRISMA-ScR: Preferred Reporting Items for Systematic Reviews and Meta-Analyses Extension for Scoping Reviews
SMC: secure multiparty computation
