# Supplementary material for: Applications of Federated Learning in Mobile Health: Scoping Review
Source: J Med Internet Res. 2023 May 1;25:e43006. doi: 10.2196/43006 (PMC10186185; doi:10.2196/43006)
Supplement: Multimedia Appendix 1 [file jmir_v25i1e43006_app1.docx]

Table 1. Search strategy and search results per search engine

| Database | Initial Search | Second Search | Total |
| --- | --- | --- | --- |
| PubMed | ("federated learning" AND "mobile" AND "health*") AND (("2016/01"[Date - Publication]: "2022/01"[Date - Publication])) | ("collaborative learning" AND "mobile" AND "health*") AND (("2015/01"[Date - Publication]: "2018/01"[Date - Publication])) |  |
|  | 4 | 3 | 7 |
| IEEE Xplore | ("All Metadata":"federated learning") AND ("All Metadata":"mobile") AND ("All Metadata":"health*")  2016-present | ("All Metadata":"collaborative learning") AND ("All Metadata":"mobile") AND ("All Metadata":"health*")  2015-2018 |  |
|  | 31 | 4 | 35 |
| ACM Digital Library | [All: "federated learning"] AND [All: "mobile"] AND [All: "health*"] AND [E-Publication Date: (01/01/2016 TO present)] | [All: "collaborative learning"] AND [All: "mobile"] AND [All: "health*"] AND [E-Publication Date: (01/01/2015 TO 01/01/2018)] - “Research Article” |  |
|  | 224 | 102 | 326 |
| Web of Science | (ALL=("federated learning" AND "mobile" AND "health*")) AND DOP=(2016-01-01/present) | (ALL=("collaborative learning" AND mobile AND health*)) AND DOP=(2015-01-01/2018-01-31) |  |
|  | 16 | 2 | 18 |
| JMIR | "federated learning" AND "mobile" AND "health" within 2016/01-present |  |  |
|  | 8 |  | 8 |
| ScienceDirect | "federated learning" AND "mobile" AND "health"  Year: 2016-present |  |  |
|  | 192 |  | 192 |
| Springer | "federated learning" AND mobile AND health*  within 2016-present |  |  |
|  | 509 |  | 509 |
| Total | 984 | 111 | 1095 |

*** Note:** The term “present” in this context refers to mid-January 2022, at which point the search process was conducted. And the search results are valid as of that date. A supplementary search for “collaborative learning” was also conducted, but it should be noted that using this term in the ACM digital library resulted in a large number of irrelevant studies, as it is a commonly used term in the field of education. To address this, a content-type screening was implemented immediately upon obtaining the results in the second search.
