# Supplementary material for: Applications of Federated Learning in Mobile Health: Scoping Review
Source: J Med Internet Res. 2023 May 1;25:e43006. doi: 10.2196/43006 (PMC10186185; doi:10.2196/43006)
Supplement: Multimedia Appendix 2 [file jmir_v25i1e43006_app2.docx]

Table 2. Summary of datasets used in the included studies

| **Datasets** | **Type of data** | **Labels** | **Studies** |
| --- | --- | --- | --- |
| Human Activity Recognition Using Smartphones | Acceleration, angular velocity | Walking, walking upstairs, walking downstairs, sitting, standing, laying | ADLs [12,32-34,37,38] |
| Heterogeneity Human Activity Recognition | Acceleration, angular velocity | Biking, sitting, standing, walking, stairs up, stairs down | ADLs [4,31] |
| MHEALTH Dataset | Acceleration, angular velocity | Standing still,  sitting and relaxing, lying down,  walking,  climbing stairs,  waist bends forward,  frontal elevation of arms,  knees bending (crouching), cycling,  jogging, running,  jump front & back | ADLs [33]  Perioperative complications prognostic prediction [29] |
| WISDM | Acceleration, angular velocity | Walking, jogging, stairs, sitting, standing, typing, brushing teeth, eating soup, eating chips, eating pasta, drinking from cup, eating sandwich, kicking (soccer ball), playing catch w/tennis ball, dribbling (basketball), writing, clapping, folding clothes | ADLs [37] |
| RealWorld (HAR) | Acceleration, GPS, angular velocity, light, magnetic field, and sound level | Walking, sitting, standing, running, lying, stairs up, stairs down, jumping | ADLs [30,38] |
| USC-HAD | Acceleration, angular velocity | Walking forward, walking left, walking right, walking upstairs, walking downstairs, running forward, jumping, sitting, standing, sleeping, elevator up, elevator down | ADLs [4] |
| Li et al. | Acceleration, angular velocity | Walking, biking, upstairs,  downstairs, running and taking the bus | ADLs [4] |
| PAMAP2 | Acceleration, angular velocity, magnetometer, orientation, temperature, heart rate | Lying, sitting, standing, walking, running, cycling, Nordic walking, watching TV, computer work, car driving, ascending stairs, descending stairs, vacuum cleaning, ironing, folding laundry, house cleaning, playing soccer, rope jumping | ADLs [13,37] |
| MobiAct | Acceleration, angular velocity, orientation | Standing, walking, stairs up, stairs down, jumping, jogging, car step in, car step out, sit chair, fall | ADLs [36] |
| OPPORTUNITY | Acceleration, rate of turn, magnetic field, orientation | Relaxing, early morning, coffee time, sandwich time, clean up, open then close the fridge,  open then close the dishwasher,  open then close drawers,  open then close door, toggle the lights on then off,  clean the table, drink while standing, drink while seated | ADLs [13,37] |
| HARBox | IMU data | Walking, hopping, phone calls, waving, typing | ADLs [33,34] |
| IMU Dataset | IMU data | Walking on corridors/ upstairs/ downstairs | ADLs [34] |
| UWB Dataset | Distance by two-way ranging | With/ without human movement | ADLs [34,35] |
| Depth Dataset | ROI (region of interest) of the depth gesture | Good, okay, victory, stop, fist | ADLs [34,35] |
| Chen et al. | Acceleration, angular velocity | Arm droop, balance,  gait, postural tremor, resting tremor | Parkinson’s disease monitoring [12] |
| Daphnet Freezing of Gait Dataset | Acceleration for Freezing of Gait | Walking straight, random walking, entering and leaving rooms, walking  to the kitchen, getting something to drink,  returning to the starting room with the cup of water | Parkinson’s disease monitoring [13] |
| WESAD | Blood volume pulse (BVP), electrocardiogram (ECG), electrodermal activity (EDA), electromyogram (EMG), respiration, body temperature, acceleration | Affective states—neutral, stress, amusement | Stress monitoring [18] |
| SWELL | Heart rate (variability), skin conductance, body postures, facial expressions, computer interactions | Task load (NASA-TLX), Mental effort (RSME), Emotion (SAM), Perceived stress | Stress monitoring [16] |
| The Ford Challenge Dataset | Physiological, environmental, and vehicular data | Alert/ not alert (driver status) | Stress monitoring [16] |
| Can et al. | Heart activity, electrodermal activity, acceleration | Low stress, high stress | Stress monitoring [17] |
| Xu et al. | Alphanumeric characters, special characters, accelerometer value | Bipolar I disorder, bipolar II disorder, normal | Depression detection [9] |
| Fang et al. | Exercise time, running speed | Heart rate | Cardiac health monitoring [19] |
| Physical Activity Recognition Dataset | Acceleration | Running, slow-walk, fast-walk, aerobic dancing, stairs up and stairs down | Cardiac health monitoring [20] |
| MIT-BIH Arrhythmia Database | ECG | Uknown | Cardiac health monitoring [21] |
| Atlas Dermatology dataset | Images of dermatology diseases | Different categories of skin disease | Skin disease diagnosis [26,27] |
| Dermnet Dataset | Images of dermatology diseases | Different categories of skin disease | Skin disease diagnosis [27] |
| ISIC 2019 Challenge dataset | Images of dermatology diseases | Different categories of skin disease | Skin disease diagnosis [27] |
| Breast Cancer Wisconsin Dataset | Digitized image of a fine needle aspirate (FNA) of a breast mass | Benign, malignant | Breast cancer [28] |
| Siddiqui et al. | Frequency of collecting pulse rate, temperature, blood pressure, weight and height | Extremely weak, weak, normal, overweight, obesity, extreme obesity | Risk of obesity [23] |
| Pima Indians Diabetes Dataset | Glucose, diastolic blood pressure, triceps skin fold thickness, BMI, insulin, number of pregnancies, diabetes pedigree function, age | Diabetes, not diabetes | Diabetes management [22] |
| Dataset I of the Brain-Computer  Interface Competition III (BCI-III-I) | Electrocorticography (ECoG) of epileptic patients | Epileptic positive, negative | Epilepsy [15] |
| Dataset from International Conference on  Machine Learning | Psychological data and characteristics of subjects | Positive, negative | Epilepsy [15] |
| PLAGH Dataset | Patient’s basic information, biochemical data, and review  scale | With delirium, without delirium | Perioperative complications prognostic prediction [29] |
