# Supplementary material for: Applications of Federated Learning in Mobile Health: Scoping Review
Source: J Med Internet Res. 2023 May 1;25:e43006. doi: 10.2196/43006 (PMC10186185; doi:10.2196/43006)
Supplement: Multimedia Appendix 3 [file jmir_v25i1e43006_app3.docx]

Table 3. Summary of the included studies

| ­Author (Year) | Real-world Applications | Data | Challenges | Proposed Approach |
| --- | --- | --- | --- | --- |
| Li et al.  (2021)  [4] | Remote monitoring — ADLs | Acceleration, angular velocity | Statistical heterogeneity, expensive communication | A federated representation learning framework, in which an embedding network is meta-learned in a federated learning way. |
| Xu et al.  (2021)  [9] | Diagnostic and treatment support — depression | Alphanumeric characters, special characters, accelerometer value | Expensive communication, real-time data stream | A multi-view FL framework using multi-source real-time data collected from smartphone keyboards. |
| Chen et al.  (2020)  [12] | Remote monitoring — Parkinson’s disease | Acceleration, angular velocity | Statistical heterogeneity, expensive communication, privacy leakage | A federated transfer learning framework that can achieve relatively personalized model learning through knowledge transfer. |
| Zhao et al.  (2020)  [13] | Remote monitoring — Parkinson’s disease | Acceleration, angular velocity, magnetometer, orientation, temperature, heart rate, rate of turn, acceleration for freezing of gait | Expensive communication | A system built on top of the Databox platform for personal data management and access control. |
| Gong et al.  (2016)  [15] | Remote monitoring — Epilepsy | ECoG, psychological data and characteristics of subjects | Expensive communication, privacy leakage | A private scheme based on alternating direction method of multipliers (ADMM). |
| Zhang et al.  (2021)  [16] | Remote monitoring — stress | Heart rate (variability), skin conductance, body postures, facial expressions, computer interactions, physiological, environmental, and vehicular data | Statistical heterogeneity, system heterogeneity, privacy leakage | A new local update scheme and an adaptive global update scheme, and these two components jointly allow each device to decide the optimized local and global update strategy. |
| Can et al. (2021)  [17] | Remote monitoring — stress | Heart activity, electrodermal activity, acceleration | Scalability | A federated learning framework on the heart activity data. |
| Liu et al.  (2021)  [18] | Remote monitoring — stress | BVP, ECG, EDA, EMG, respiration, body temperature, acceleration | Statistical heterogeneity | Add a simple user embedding to their neural network for collaborative personalization in FL. |
| Fang et al.  (2021)  [19] | Remote monitoring — — cardiac health | Exercise time, running speed | None | A Bayesian model along with the distributed inference algorithm. |
| Ogbuabor et al.  (2021)  [20] | Remote monitoring — cardiac health | Acceleration | Expensive communication, scalability | A context-aware decision support system for cardiac condition monitoring during rehabilitation. |
| Raza et al.  (2022)  [21] | Remote monitoring — cardiac health | ECG | Statistical heterogeneity, expensive communication, privacy leakage, real-time data stream, data scarcity | A privacy-preserving, efficient and interpretable/explainable AI-based end-to-end framework for EEG signal classification. |
| Gong et al.  (2015)  [22] | Remote monitoring — Diabetes | Glucose, diastolic blood pressure, triceps skin fold thickness, BMI, insulin, number of pregnancies, diabetes pedigree function, age | Privacy leakage, data scarcity | A scheme that keeps the training samples private based on alternating direction method of multipliers (ADMM). |
| Siddiqui et al. (2021)  [23] | Remote monitoring — obesity | Frequency of collecting pulse rate, temperature, blood pressure, weight and height | Real-time data stream | IoMT architecture integrated with federated learning model for managing obesity risk remotely in real-time. |
| Elayan et al. (2021)  [26] | Diagnostic and treatment support — dermatological disease | Images of dermatology diseases | Expensive communication | A federated learning framework for decentralized healthcare systems with an algorithm for an automated training data acquiring process. |
| Wu et al.  (2021)  [27] | Diagnostic and treatment support — dermatological disease | Images of dermatology diseases | Expensive communication | An on-device federated contrastive learning framework to enable effective learning with limited labels for dermatological disease diagnosis. |
| Guo et al.  (2020)  [28] | Diagnostic and treatment support — breast cancer | Digitized image of a fine needle aspirate of a breast mass | Expensive communication, privacy leakage | A federated edge learning system which can process the distributed private data in parallel with a two-stage differential privacy scheme. |
| Sun et al.  (2021)  [29] | Diagnostic and treatment support — perioperative complications | Patient’s basic information, biochemical data, and review scale, acceleration, angular velocity | Privacy leakage | A federated learning framework which bridges Inner- and Outer-hospital information for perioperative complications prognostic prediction. |
| Ek et al. (2020)  [30] | Remote monitoring — ADLs | Acceleration, GPS, angular velocity, light, magnetic field, and sound level | Statistical heterogeneity, expensive communication | Three main FL algorithms were implemented and evaluated on the HAR tasks. |
| Gudur et al.  (2021)  [31] | Remote monitoring — ADLs | Acceleration, angular velocity | Statistical heterogeneity | A framework with two different versions for federated aggregation, which leverages overlapping information gain across activities. |
| Liu et al.  (2016)  [32] | Remote monitoring — ADLs | Acceleration, angular velocity | Expensive communication | A collaborative privacy-preserving learning system that is implemented on a server and several mobile devices. |
| Lyu et al.  (2017)  [33] | Remote monitoring — ADLs | Acceleration, angular velocity, IMU data | Privacy leakage, data scarcity, scalability | A two-stage privacy-preserving scheme for privacy-preserving collaborative deep learning. |
| Ouyang et al.  (2021)  [34] | Remote monitoring — ADLs | Distance by two-way ranging, ROI of the depth gesture, IMU data | Statistical heterogeneity, expensive communication, system heterogeneity, data scarcity | A similarity-aware federated learning system enabling collaborative learning among similar nodes and integrates two effective communication optimization mechanisms based on the learned cluster structure. |
| Tu et al. (2021)  [35] | Remote monitoring — ADLs | Acceleration, angular velocity, distance by two-way ranging, ROI of the depth gesture | Statistical heterogeneity,  expensive communication, system heterogeneity, real-time data stream | A novel federated deep learning system for HAR that captures the similarity of users’ models and learn personalized models through dynamic layer sharing in an iterative layer-wise manner. |
| Wu et al.  (2020)  [36] | Remote monitoring — ADLs | Acceleration, angular velocity, orientation | Statistical heterogeneity, expensive communication, real-time data stream | A cloud-edge based federated learning framework for in-home health monitoring, which learns a shared global model in the cloud from multiple homes at the network edges with a GCAE. |
| Xiao et al.  (2021)  [37] | Remote monitoring — ADLs | Acceleration, angular velocity, magnetometer, orientation, temperature, heart rate | Statistical heterogeneity, expensive communication, privacy leakage | A secure federated learning system that adopts a perceptive extraction network as the feature extractor for each user. |
| Yu et al.  (2021)  [38] | Remote monitoring — ADLs | Acceleration, GPS, angular velocity, light, magnetic field, and sound level | Statistical heterogeneity, expensive communication, privacy leakage, real-time data stream, scalability | A personalized federated HAR framework based on semi-supervised online learning with a hierarchical attention architecture for the alignment of different level features. |

**Abbreviations**:

ADLs= Activities of daily living, BMI=Body mass index, BVP=Blood volume pulse, ECG=Electrocardiogram, ECoG=Electrocorticography, EDA=Electrodermal activity, EMG=Electromyogram, FL=Federated learning, GCAE=Generative convolutional autoencoder, HAR=Human activity recognition, IMU=Inertial measurement unit, ROI=Region of interest.
